# Zinc monotherapy is effective in Wilson’s disease patients with mild liver disease diagnosed in childhood: a retrospective study

**DOI:** 10.1186/1750-1172-9-41

**Published:** 2014-03-25

**Authors:** Giusy Ranucci, Fabiola Di Dato, Maria Immacolata Spagnuolo, Pietro Vajro, Raffaele Iorio

**Affiliations:** 1 Department of Translational Medical Science, Section of Pediatrics, University of Naples Federico II, Via Pansini 5, 80131 Naples, Italy; 2 Chair of Pediatrics, Department of Medicine and Science, University of Salerno, 84081 Fisciano (Salerno), Italy

**Keywords:** D-penicillamine, Zinc, Kayser-Fleischer, Copper, ATP7B, Children, Liver disease

## Abstract

**Background:**

Wilson’s disease (WD) evolves rapidly and is fatal if untreated. The treatment of WD patients with mild liver disease is not clearly defined. To address this issue, we evaluated long-term outcomes of three treatment regimens (D-penicillamine, zinc or both) in patients diagnosed in childhood.

**Methods:**

We retrospectively evaluated efficacy, compliance and reasons for treatment discontinuation in 42 WD patients (median age at diagnosis: 6 years; median follow-up: 12 years) with mild liver disease. Treatment duration for each treatment block until a medication change or completion of follow-up was analyzed. Events of change of treatment were evaluated using Kaplan-Meier analysis.

**Results:**

Total discontinuations due to treatment failure or adverse events were more frequent in patients receiving D-penicillamine (45%) or combination (36%) therapy than in patients receiving zinc (12%) (P = .001 and P = .02, respectively). Treatment failure was more frequent on D-penicillamine (28%) and combination therapy (36%) than on zinc (12%); the difference was statistically significant only between zinc and combination therapy (P = .03). First-line zinc monotherapy controlled WD-related liver disease in 13/15 patients (87%); the two subjects that failed on zinc were poor adherent. Zinc was effective in 3/5 (60%) patients that failed on D-penicillamine and combination regimens. All 15 D-penicillamine responders that switched to zinc had good control of liver disease at a median follow-up of 13.1 years. Among 6 D-penicillamine non-responders that switched to zinc, 4 (67%) responded. At follow-up completion, only 5/42 (12%) patients failed. Adverse event-induced discontinuation was significantly more frequent in patients on D-penicillamine than in patients receiving zinc (P = .03).

**Conclusions:**

Zinc monotherapy is effective in controlling WD-related liver disease both as first-line and as maintenance treatment in patients with mild liver disease diagnosed in childhood.

## Background

Wilson’s disease (WD) is an autosomal-recessive disorder caused by mutations in the *ATP7B* gene [1]. Clinical presentation can vary widely [2,3], but the key features of WD are liver disease, neuropsychiatric disturbances, Kayser–Fleischer (KF) rings, and acute episodes of hemolysis often in association with acute liver failure. WD presents with liver disease more often in children and young adults than in older adults [4]. An early diagnosis is important because WD can be fatal if left untreated. Once the diagnosis has been made [2,5], treatment is a lifelong necessity, and compliance must be zealously monitored [6].

The approach to treatment (drug of choice and dosage) depends on clinical presentation at diagnosis, and the severity of neurologic or hepatic disease [6]. Current treatment regimens include chelators (D-penicillamine and trientine) that promote the excretion of copper from the body, and/or zinc salts that reduce copper absorption [2,4].The best therapeutic approach for each specific presentation of the disease remains controversial and there are no clear indications about how to treat WD patients with a mild liver disease [2,4]. Transplantation remains the treatment of choice for patients with acute liver failure due to WD as well as for treatment of patients with chronic liver failure unresponsive to medical therapy [2]. In patients with severe neurological presentation the use of zinc seems to be preferred [6].

Studies conducted on distinct groups of WD patients have demonstrated that zinc monotherapy is safe and inexpensive [7]. Zinc was successfully used as maintenance therapy in presymptomatic and pregnant patients; as first-line treatment in patients with neurological onset and in WD children with hepatic presentation [8-16]. Somewhat in contrast with these findings, Weiss et al. questioned the efficacy of zinc therapy in WD based on the retrospective observation that, in adult patients, zinc monotherapy was less effective than chelating agents in preventing the progression of liver disease and in terms of survival in the absence of liver transplantation [17].

In the attempt to provide indications regarding the treatment of WD patients with mild liver disease, and to shed light on the discordance between evidence supporting the efficacy of zinc treatment and the studies against this therapy [17,18], we evaluated the long-term response to therapy and particularly the efficacy of zinc monotherapy in a series of WD patients with mild liver disease diagnosed in childhood.

## Patients and methods

### Patients

We retrospectively evaluated the records of all patients with WD referred to Liver Unit of our Department of Pediatrics between 1984 and 2012. Patients were eligible for the study if: 1) the diagnosis of WD was confirmed by molecular analysis or liver copper content >250 μg/g dry tissue or both; 2) WD was diagnosed in childhood; and 3) data were available about clinical-laboratory features, copper metabolism and treatment (dosage and compliance) at the time of diagnosis and throughout the period of observation.

### Analysis of treatment

We evaluated the clinical, laboratory and sonographic features of all patients at diagnosis of WD and throughout the observation period. Patients were considered to be affected by mild liver disease in the presence of one of the following features: (1) abnormalities of liver enzymes (aspartate aminotransferase [AST], alanine aminotransferase [ALT], γ-glutamyltransferase [GGT]) not associated to symptoms of liver disease (fatigue, anorexia, jaundice, ascites, pruritus, palmar erythema, clubbing, spider nevi) and with normal liver function tests, namely albumin, prothrombine time, bilirubin; (2) hepatomegaly with sonographic evidence of fatty liver and/or liver echostructure dishomogeneity also with normal values of liver enzymes.

We also recorded data about treatment administered in each patient during follow-up. Specifically, we collected information about each drug used, doses, efficacy, side-effects, and reasons for medication changes. We classified the treatment regimen as: (1) D-penicillamine, (2) zinc salts, and (3) a combination of D-penicillamine and zinc. Trientine was not included because it was not commercially available in Italy at the time of the study.

We recorded the reasons for discontinuation of a drug as: treatment failure, adverse event(s), patient’s request (not linked to adverse events) and maintenance regimen. Medication changes due to a shift to maintenance therapy established by the physician or upon the patient’s request in the absence of adverse events were not considered in the analysis of adherence to treatment. The duration of each treatment block was defined as the period between treatment onset and either a change of medication, or completion of follow-up. Treatment blocks lasting < 6 months were not included in the analysis.

The main measure of effectiveness was the impact of therapy on the concentrations of liver enzymes (AST, ALT, GGT). Liver treatment failure was diagnosed in case of persistent hypertransaminasemia and/or in case of appearance of new clinical and/or laboratory and/or sonographic signs of disease and/or in case of the need of liver transplantation and/or death.

Patients were classified as responder or non-responder. Specifically, responders were patients whose liver enzyme values were significantly lower than baseline levels, and whose AST, ALT and GGT levels were at least lower than twice the upper limit of normal at the end of a treatment block. Patients with normal baseline levels of liver enzymes were considered responders if they maintained normal values and if other clinical, laboratory and sonographic parameters remained unchanged during follow-up. Patients were considered relapsers when efficacy parameters became deranged after a favorable response. At the end of the study, relapsers were included in the non-responder group if inadequate dosage and other intercurrent causes of liver disease were excluded. In case of non-response to therapy, we looked for signs of poor adherence, inadequate dosage and other intercurrent causes of liver disease.

Adherence to treatment was assessed based on data about the treatment schedule (prescribed dose, number of daily doses, and adequate interval between medicine and meals), and about levels of urinary copper excretion (<75 μg/24 h), urinary zinc levels (> 2000 μg/24 h) and serum zinc levels (> 150 mg/dl) for patients treated with zinc, and urinary copper levels (values between 200 and 500 μg/24 h after a year of treatment) for patients treated with D-penicillamine.

Neurological worsening in WD patients was assessed by an oriented examination of central nervous system using clinical and radiological investigations if required.

## Methods

As recommended [2,4], D-penicillamine was used as initial and maintenance therapy for symptomatic patients, zinc salts were used as first-line treatment and maintenance for presymptomatic patients, and as maintenance therapy after a first phase with penicillamine for symptomatic patients. Starting from 1995, zinc was also used as first-line therapy in WD patients with a mild liver disease.

D-penicillamine was used as initial therapy at the dosage of 20 mg/kg daily divided in two to four doses; the maintenance dose was between 750–1500 mg daily in two or three doses. Zinc was given in sulphate form until 2004, and subsequently, when licensed in Italy, as acetate at the dosage of elemental zinc 25 mg twice daily in children younger than 6 years; 25 mg three times a day for children aged between 6 and 16 years of age (or weight <57 kg); and 50 mg three times daily in patients older than 16 years of age (or weight >57 kg). Patients were instructed to take their medication at least one hour before meals or at least 2 hours after meals. The study complies with the Declaration of Helsinki.

### Statistical analysis

Events of change of treatment were evaluated using Kaplan-Meier analysis. We established P values for this calculation using the pairwise log-rank test (Mantel-Cox). All P values were based on two-tailed comparisons, and those less than 0.05 were considered to indicate statistical significance. All statistical analyses were performed with GraphPad Prism version 6.00 for Mac (GraphPad Software, San Diego, CA).

## Results

### Initial presentation of the study group

Forty-eight consecutive patients (35 males) with a diagnosis of WD were evaluated, and 42 (30 males, median age at diagnosis: 6 years, range: 1–16 years) were selected for treatment analysis based on our entry criteria. The *ATP7B* gene was analyzed in 39 patients, and disease-causing mutations were found in 37 (17 compound heterozygotes, 13 homozygotes and 7 with a single known mutation). Nine patients were referred for family screening. All patients had signs of mild liver disease: 39 presented abnormal liver enzymes. At diagnosis, ultrasound showed hepatomegaly in 40/42 patients (95%) and steatosis in 39/42 patients (93%). Three patients had steatosis on ultrasound and normal basal liver enzymes.

In 4 patients (9.5%), concomitant neurological/psychiatric signs (tremor, micrography, behavioral disorders) were identified only after a detailed neurological examination at the time of diagnosis, and KF rings were detected in 2 of these 4 patients. KF rings were not present in any of the remaining patients. In this subset of 4 patients brain magnetic resonance at diagnosis showed no structural abnormalities in the basal ganglia.

Baseline characteristics of 42 patients are reported in Table 1.

**Table 1:** Baseline characteristics of 42 patients with WD

**Patient**	**Sex**	**Age at diagnosis (months)**	**Diagnosis by familial screening**	**Presentation**	**ATP7B genotype**	**AST (UI/L)**	**ALT (UI/L)**	**GGT (UI/L)**	**Ceruloplasmin (mg/dl)**	**Basal urinary copper (mcg/24 h)**	**Hepatic copper (mcg/g dry weight)**	**Liver biopsy**
1	F	48	Yes	Hepatic	No mutation found	168	210	21	8	4	1060	F (IS:1)
2	M	96	No	Hepatic	No mutation found	30	63	11	5	14	250	F (IS:1)
3	M	108	No	Hepatic	p.H1069Q/p.H1069Q	78	202	78	23	198	750	S, F (IS:1)
4	F	78	No	Hepatic	p.H1069Q/unknown	72	196	38	15	180	514	S, F (IS:1)
5	M	101	No	Hepatic	p.H1069Q/p.H1069Q	112	252	70	19	120	ND	ND
6	M	60	No	Hepatic	p.T858A/c.51 + 4A > T	179	514	99	3	139	532	S, F (IS: 2)
7	F	16	Yes	Hepatic	p.P840L/p.N1270S	53	28	20	3	15	ND	ND
8	M	87	No	Hepatic	p.P840L/p.N1270S	96	288	77	2	135	919	S, F (IS:2), ACH
9	F	13	Yes	Hepatic	c.2299insG/c.2299insG	42	27	16	7	ND	ND	ND
10	M	19	Yes	Hepatic	c.2299insG/c.2299insG	67	35	15	6	15	ND	ND
11	M	170	No	Hepatic	c.51 + 384_1708-953del/ c.51 + 384_1708-953del	199	582	267	2	254	ND	S, F (IS:1)
12	M	125	No	Mixed	p.H1069Q/p.H1207P	35	106	62	18	300	300	F (IS: 2)
13	M	74	No	Hepatic	p.R1319X/p.R1319X	161	362	101	2	116	260	S, F (IS:2)
14	F	80	No	Hepatic	p.P840L/p.N1270S	153	370	80	2	117	ND	F (IS: 2)
15	M	52	No	Hepatic	p.R1319X/unknown	137	307	55	2	40	1203	S, F (IS:1)
16	M	12	Yes	Hepatic	c.2447 + 5A > G/c.2447 + 5A > G	55	57	20	11	ND	ND	ND
17	M	73	No	Hepatic	c.2447 + 5A > G/c.2447 + 5A > G	114	148	51	8	262	1056	S, F (IS: 2)
18	M	58	No	Hepatic	c.2447 + 5A > G/c.2447 + 5A > G	150	424	47	6	241	1048	S (IS:1)
19	M	72	No	Hepatic	p.H1069Q/p.T1220M	117	412	70	12	228	ND	S, F (IS:2)
20	M	36	Yes	Hepatic	p.H1069Q/p.T1220M	22	12	13	16	31	ND	ND
21	M	64	No	Hepatic	p.D765N/unknown	99	201	105	6	137	407	ND
22	M	65	No	Hepatic	p.H1069Q/p.H1069Q	130	395	187	16	270	1129	S, F (IS: 1)
23	F	79	No	Mixed	p.T1220M/c.51 + 4A > T	64	208	61	2	119	1041	S, F (IS: 1)
24	F	29	Yes	Hepatic	p.T1220M/c.51 + 4A > T	54	50	21	2	22	1002	S, F (IS: 1)
25	F	92	No	Hepatic	p.H1069Q/p.R1041P	52	110	36	18	108	714	S, F (IS: 1)
26	M	130	No	Hepatic	p.T1220M/unknown	100	148	100	3	84	1060	S, ACH
27	M	152	No	Hepatic	p.T1220M/unknown	33	52	73	2	413	60	S, ACH
28	F	70	No	Hepatic	ND	205	398	83	7	172	676	S, F (IS:1)
29	M	100	No	Hepatic	p.S1369L/p.S1369L	102	173	60	2	236	ND	S, F (IS:2)
30	M	76	No	Hepatic	p.G591D/p.R969Q	146	502	119	7	780	761	S
31	M	69	No	Hepatic	c.2304-2305 insC/c.2304-2305 insC	117	276	103	2	595	977	S
32	M	42	No	Hepatic	p.V1262F/p.V1297N	117	262	41	2	14	ND	ND
33	M	73	No	Hepatic	p.H1069Q/p.G711E	169	480	227	18	150	620	S, F (IS: 1)
34	M	192	No	Mixed	p.H1069Q/p.N1270S	27	23	32	6	165	970	S, F (IS: 2)
35	M	75	No	Hepatic	c.1782delT/c.3182G > A	185	398	70	4	180	296	S, F (IS: 2)
36	M	29	Yes	Hepatic	c.1782delT/c.3182G > A	69	64	16	2	16	1230	S
37	F	63	No	Hepatic	p.A1003T/p.A1003T	80	147	45	38	66	785	S, ACH
38	M	99	No	Hepatic	ND	79	181	89	4	91	780	S, F (IS 1)
39	F	72	No	Hepatic	p.I899F/p.N1270S	66	215	67	8	157	1449	S, F (IS: 1)
40	M	56	No	Hepatic	c.2304dupC/unknown	188	483	102	2	138	1660	S, F (IS: 2)
41	M	39	Yes	Hepatic	c.2304dupC/unknown	51	37	11	3	25	904	S, F (IS:1)
42	F	84	No	Mixed	ND	156	187	191	3	145	600	S, F (IS: 1)

*Abbreviations:* IS: Ishak Fibrosis Score; ACH, active chronic hepatitis; F, fibrosis; ND, not done; S, steatosis.

### Treatment type in the study groups

Patients were first treated with D-penicillamine (n = 27) or zinc (n = 15). Of the 27 patients first treated with D-penicillamine (median follow-up: 13.3 years; range: 6.7-25.2 years), 19 (70%) shifted to zinc monotherapy. Of the 15 patients initially treated with zinc (median follow-up: 9.3 years; range: 1.6-19.8 years), 13 (86%) continued zinc monotherapy, 1 (7%) returned to zinc after 3 years of combination therapy started because of relapse on initial treatment, and 1 (7%) shifted to combination therapy. At the end of follow-up (median duration: 12 years; range: 1.6-25.2 years), 33 (79%) were on zinc, 8 (19%) on D-penicillamine and 1 (2%) on combination therapy. Basal features of two groups initially treated with D-penicillamine or zinc are reported in Table 2.

**Table 2:** Basal clinical and laboratory features of patients initially treated with D-penicillamine or zinc

	**D-Penicillamine**	**Zinc**	**P values**
Patients (N)	27 (21 males)	15 (9 males)	
Median age at diagnosis (months)	75 (range 39–192)	42 (range 12–101)	<0.001*
Median ALT at diagnosis (UI/L)	251 (range 23–582)	110 (range 12–502)	0.022*
Median AST at diagnosis (UI/L)	114 (range 27–205)	67 (range 22–168)	0.059 (NS)
Median GGT at diagnosis (UI/L)	72 (range 11–267)	21 (range 13–119)	0.019*
Basal urinary copper (μg/24 h)	150 (range 14–413)	31 (range 4–780)	0.91 (NS)
Median liver copper content (μg/g dry tissue)	780 (range 60–1660)	989 (range 407–1230)	0.423 (NS)

*P values less than 0.05 were considered statistically significant; NS: not significant.

### Reasons for medication changes and treatment blocks

Therapy was changed in 76 treatment blocks (30 D-penicillamine therapy, 34 zinc therapy and 12 combination therapy). Of these, 74 treatment blocks (29 D-penicillamine therapy, 34 zinc therapy and 11 combination therapy) were suitable for analysis.

**Figure 1:**
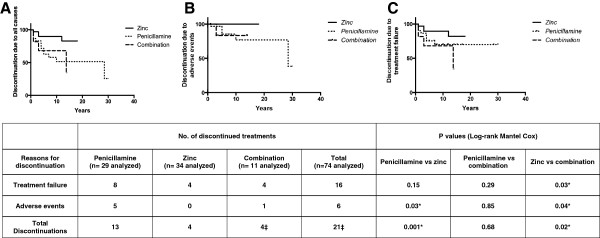
**Change of treatment due to any cause (A), adverse events (B), treatment failure (C), analyzed by the Kaplan-Meier estimation.** *P values less than 0.05 were considered statistically significant. ‡In one patient on combination therapy, discontinuation was associated with both treatment failure and adverse events.

The resulting Kaplan-Meier curve for adherence to treatment, regardless of the reasons for changing medication, is shown in Figure 1A.

A change in medication due to treatment failure or adverse events was significantly more frequent in patients receiving D-penicillamine or combination therapy than in patients receiving zinc (P = .001 and P = .02, respectively): 4/34 zinc treatment blocks (12%), 13/29 D-penicillamine blocks (45%) and 4/11 combination blocks (36%).

**Figure 2:**
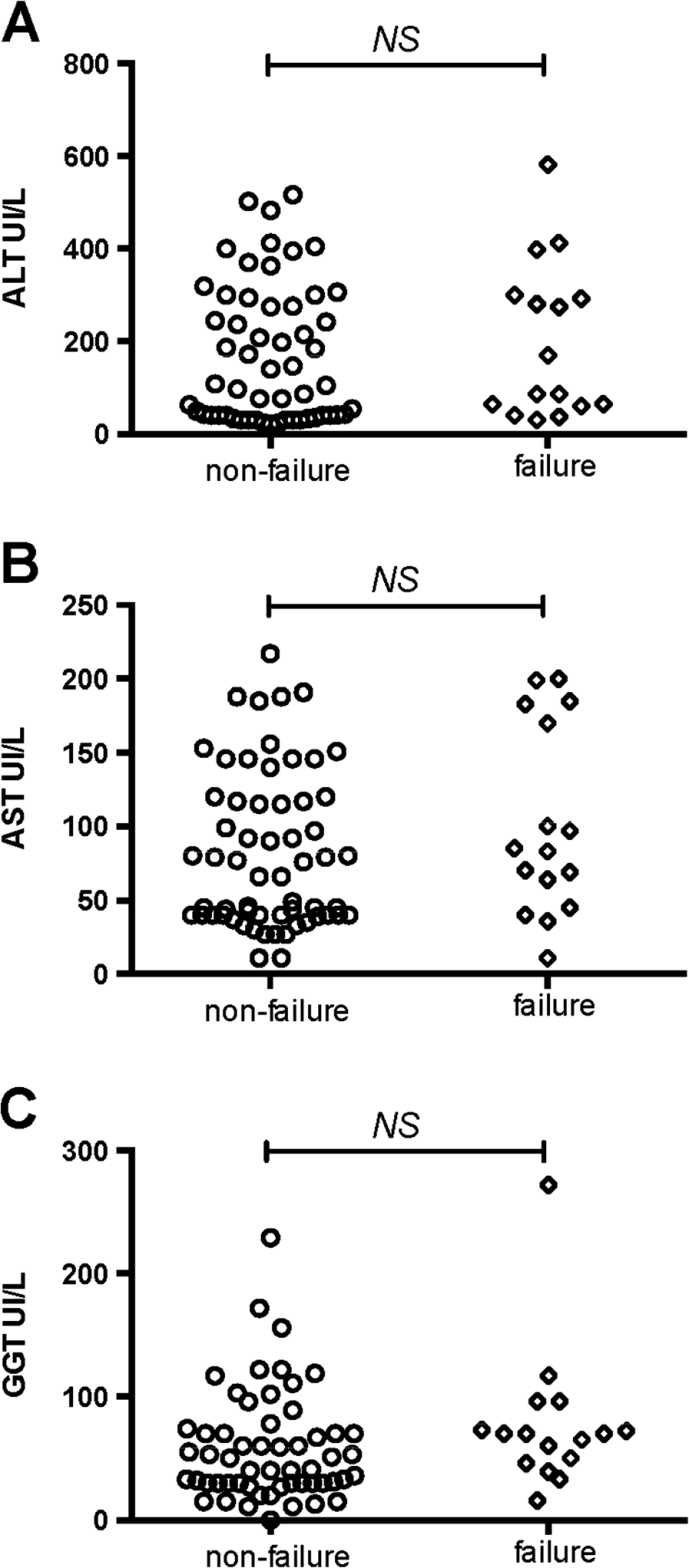
Baseline ALT (A), AST (B) and GGT (C) levels in blocks with and without treatment failure.

Change due to adverse events (Figure 1B) was significantly more frequent in patients receiving D-penicillamine and combination therapy than in patients receiving zinc (P = .03 and P = .04, respectively). In fact, 6/74 treatment blocks that included penicillamine (5 D-penicillamine and 1 combination) were stopped because of adverse events. A change in medication due to treatment failure (Figure 1C) was observed in 16 blocks (22%): 8/29 D-penicillamine blocks (28%), 4/34 zinc blocks (12%) and 4/11 combination blocks (36%). The difference was statistically significant between zinc and combination therapy (P = .03), but not between zinc and D-penicillamine (P = .15). Among treatment failure blocks, non-adherence to therapy occurred in 1/8 D-penicillamine blocks (12%), 2/4 zinc blocks (50%) and 4/4 combination blocks (100%). In one patient on combination therapy, discontinuation was associated with both treatment failure and adverse events. There were no significant differences among treatment failure versus non-failure blocks in relation to gender (P = .35) and pre-treatment liver enzyme levels (ALT, P = .50; AST, P = .21; GGT, P = .15) (Figure 2A,B and C) or fibrosis grade at diagnosis (P = .65). Zinc was effective in 3/5 patients that failed on D-penicillamine and/or combination regimen.

In 12/74 treatment blocks (16%), we recorded a transient flare-up of liver enzymes that did not lead to treatment failure in 8/34 zinc blocks (24%), after a median follow-up of 78 months (range: 36–96 months), and in 4/29 D-penicillamine treatment blocks (14%), after a median follow-up of 54 months (range: 42–72 months). Seven of the 8 relapser zinc blocks and 3/4 relapser D-penicillamine blocks were related to an inadequate dosage, and liver enzymes returned to normal value after adjusting dosage. Among relapser blocks, we identified non-adherence to therapy in 1/8 zinc blocks and in 1/4 D-penicillamine blocks.

### Response of patients to therapy

At the end of follow-up, treatment failure was recorded in 5/42 (12%) patients and 3 of these patients were poor compliers. However all patients remained symptom-free and none had worsening of liver and/or neurological disease, died or needed liver transplantation. Twenty (74%) of the 27 patients initially treated with D-penicillamine responded to therapy (within a median of 23 months; range 6–36 months). Seven of the 27 patients (26%) treated with D-penicillamine were non-responders. In this group, ALT levels decreased during treatment from a median baseline value of 398 IU/ L (173–582 IU/L) to 165 IU/L (91–377 IU/L) at the end of follow-up. One non-responder patient was treated for only 4 months and was then shifted successfully to zinc; thus, this block was not included in the discontinuation analysis.

**Figure 3:**
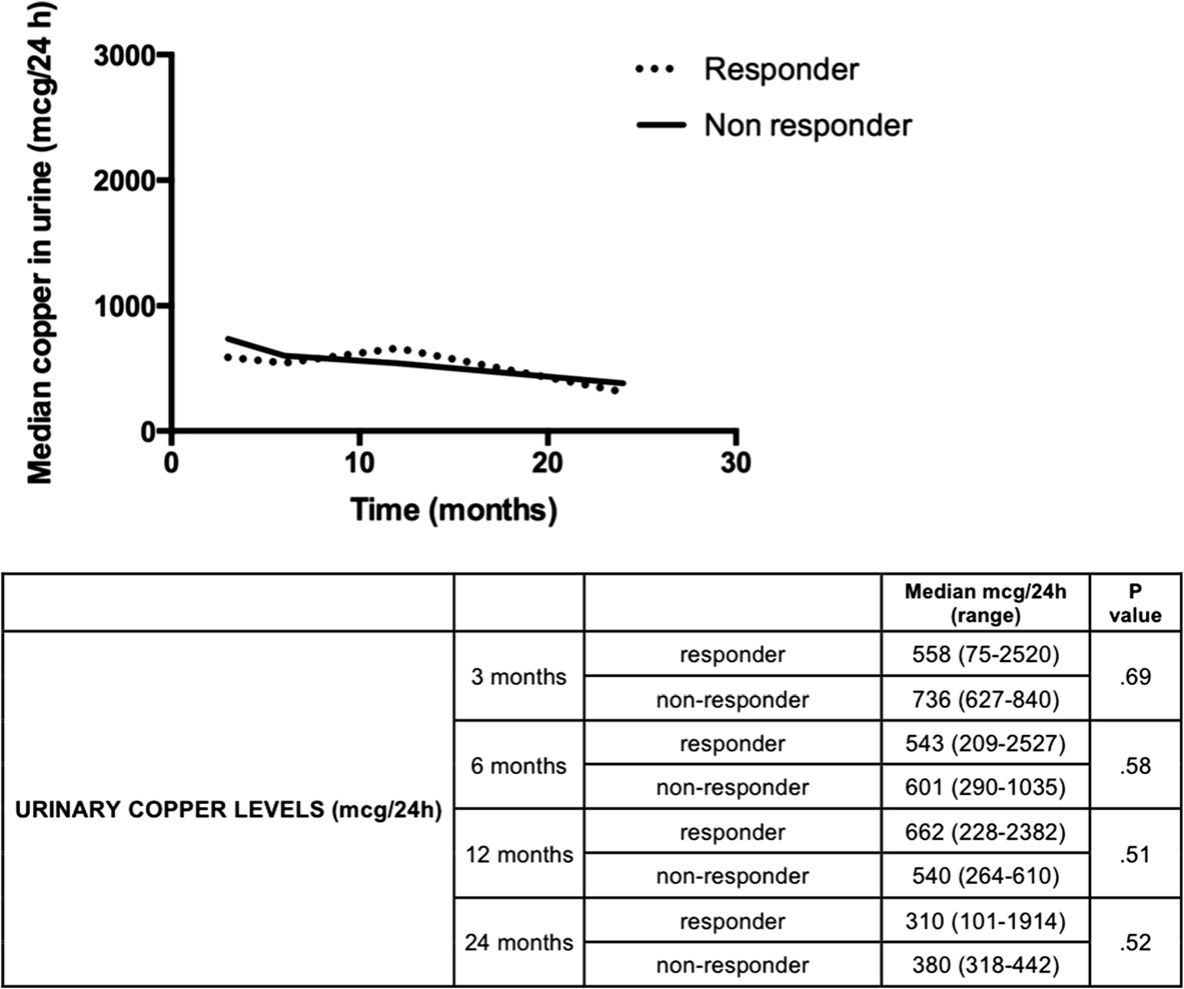
Course of urinary copper levels of patients on D-penicillamine treatment, grouped by treatment response.

Only one non-responder to D-penicillamine therapy had urinary copper excretion levels compatible with poor adherence to treatment. There were no significant differences in urinary copper excretion at 3, 6, 12 and 24 months between responders and non-responders to D-penicillamine (Figure 3). Six of the non-responders to D-penicillamine switched to zinc therapy: 4 (67%) responded to the new treatment within a median of 12 months (range 6–36 months), whereas hypertransaminasemia persisted in the other two. Among the 20 long-term responders to D-penicillamine, 6 (33%) continued therapy with this drug and obtained good control of hepatic disease (median ALT at the end of follow-up: 36 IU/L; range 12–77 IU/L), whereas 14 patients (69%) switched to zinc monotherapy (9 as maintenance therapy and 5 due to D-penicillamine-related adverse events). None of the long-term responders to D-penicillamine who switched to zinc showed hepatic worsening after a median follow-up of 13.1 years (range: 0.7 to 20.2 years) and all achieved optimal control of the disease (ALT upon completion of follow-up was 33 IU/L, range: 11–57 IU/L). Table 3 shows the data about AST, ALT and GGT in patients on D-penicillamine during the follow-up, grouped by treatment response.

**Table 3:** Course of liver enzymes in patients initially treated with D-penicillamine, grouped by treatment response

			**Median UI/L (range)**	**P value**
**ALT (UI/L)**	Baseline	Responder	201 (23–514)	.19
		Non-responder	398 (173–582)	
	6 months	Responder	122 (32–576)	.26
		Non-responder	236 (165–279)	
	12 months	Responder	60 (20–178)	≤.001
		Non-responder	91 (83–166)	
	24 months	Responder	46 (14–76)	≤.001
		Non-responder	205 (65–245)	
	>36 months	Responder	36 (12–77)	≤.001
		Non-responder	165 (91–377)	
**AST(UI/L)**	Baseline	Responder	114 (27–227)	.24
		Non-responder	117 (72–205)	
	6 months	Responder	60 (26–198)	.23
		Non-responder	94 (89–115)	
	12 months	Responder	43 (22–74)	.18
		Non-responder	60 (47–60)	
	24 months	Responder	38 (24–51)	≤.001
		Non-responder	100 (85–114)	
	>36 months	Responder	32 (20–61)	.004
		Non-responder	66 (40–76)	
**GGT(UI/L)**	Baseline	Responder	68 (11–227)	.54
		Non-responder	74 (48–267)	
	6 months	Responder	39 (14–119)	.64
		Non-responder	59 (49–65)	
	12 months	Responder	27 (13–57)	.19
		Non-responder	42 (36–48)	
	24 months	Responder	24 (12–44)	.29
		Non-responder	31 (31–32)	
	>36 months	Responder	17 (12–40)	.29
		Non-responder	31 (25–41)	

Of the 15 patients initially treated with zinc, 13 (87%) responded (within a median of 12 months; range: 3–24 months) and the median ALT at the end of follow-up was 49 IU/L (range: 18–79 IU/L). The two non-responders to zinc complied poorly with treatment, as witnessed by inadequate levels of serum and urinary zinc and/or copper excretion. Both non-responders switched to combination therapy without normalization of aminotransferase and one patient asked to return to zinc therapy. In these two patients, ALT levels on zinc therapy increased from a median baseline of 57 IU/L (range: 50–64 IU/L) to a median of 172 IU/L (range: 132–212 IU/L) at the end of follow-up. No patient showed during the entire period of observation signs of neurological and/or psychiatric worsening. Table 4 shows the profile of AST, ALT and GGT levels in the responders and non-responders to zinc therapy.

**Table 4:** Course of liver enzyme levels in patients initially treated with zinc, grouped by treatment response

			**Median UI/L (range)**	**P value**
**ALT (UI/L)**	Baseline	Responder	201 (12–502)	.30
		Non-responder	57 (50–64)	
	6 months	Responder	72 (24–233)	.93
		Non-responder	82 (64–101)	
	12 months	Responder	51 (26–94)	≤.001
		Non-responder	172 (132–212)	
	24 months	Responder	41 (23-80)	-
		Non-responder	-	
	>36 months	Responder	49 (18-79)	
		Non-responder	-	
**AST (UI/L)**	Baseline	Responder	67 (22–168)	.42
		Non-responder	59 (49–69)	
	6 months	Responder	46 (34–83)	.81
		Non-responder	56 (48–64)	
	12 months	Responder	44 (35–66)	≤.001
		Non-responder	156 (92–221)	
	24 months	Responder	40 (26-74)	-
		Non-responder	-	
	>36 months	Responder	36 (19-61)	-
		Non-responder	-	
**GGT (UI/L)**	Baseline	Responder	36 (13–119)	.34
		Non-responder	18 (16–21)	
	6 months	Responder	30 (13–49)	.35
		Non-responder	40 (22–59)	
	12 months	Responder	22 (11–55)	≤.001
		Non-responder	86 (45–127)	
	24 months	Responder	18 (13-38)	-
		Non-responder	-	
	>36 months	Responder	16 (13-43)	-
		Non-responder	-	

### Adverse events under therapy

Adverse events occurred in 28/42 patients: 10 on D-penicillamine, 13 on zinc and 5 on combination therapy. Six of the 42 patients, 5 on D-penicillamine and 1 on combination regimen, changed therapy due to severe adverse events. The following D-penicillamine-related side effects were recorded: maculo-papular rash (8 cases), elastosis perforans serpiginosa (1 case), skin pigmentation (1 case), alopecia (1 case), altered taste (2 cases), proteinuria (3 cases), leucopenia and/or thrombocytopenia (3 cases). Neurologic worsening was observed in 3 patients (7%), all under D-penicillamine: tremor in 2 children and worsening of neurological/psychiatric symptoms in a patient with hepatic and neurologic features at onset. No patient on a zinc regime needed to change treatment; the only adverse effect associated with zinc was mild gastric pain and mild elevation of serum lipase and amylase without the symptoms of pancreatitis.

## Discussion

Considering the rarity of WD and the lack of clear indications on how to treat WD patients with mild liver disease [2,4], the importance of the present study lies in the fact that it evaluated zinc monotherapy in a large and homogeneous group of patients with mild liver disease due to WD, diagnosed in childhood (median age: 6 years) and monitored for a long period (median duration: 12 years). The features of our series are remarkably different from those of other pediatric reports, which in most cases included WD children with either acute or chronic symptomatic liver disease [11,17-19]. In fact, the WD patients evaluated in the present study were affected by mild liver disease. This reflects the fact that in Italy aminotransferase serum levels are measured during all routine checkups and before surgical procedures. Regarding the retrospective nature of the study, given the rarity of WD, it is virtually impossible to carry out prospective randomized controlled trials in the short-mid term to determine the optimal therapeutic schedules. In the analyzed setting, zinc monotherapy was effective both as first-line monotherapy and as maintenance treatment after initial decoppering with the chelator D-penicillamine. Changes of therapy were significantly more frequent in patients receiving D-penicillamine or combination therapy than patients receiving zinc monotherapy. Similarly, treatment failure and adverse events occurred more frequently on D-penicillamine. Interestingly, zinc failure was more frequently related to non-adherence to therapy than D-penicillamine failure.

In our study, the rate of normalization of liver enzymes was much higher in patients who started on zinc than in those who started on D-penicillamine. However, we cannot conclude that zinc monotherapy is more effective than D-penicillamine given the heterogeneity of the groups initially treated with D-penicillamine or zinc in terms of age at diagnosis and baseline ALT and GGT levels. Therefore the main message of our study is that both D-penicillamine and zinc are effective in controlling liver involvement in WD patients with mild liver disease diagnosed in childhood. It cannot be excluded that decoppering treatment when used as first drug might favor in some way the beneficial effect of zinc monotherapy in patients switching to this regimen.

In line with previous reports [7,8,14,19], the present study indicates that clinically relevant adverse events, including neurologic worsening, in some cases requiring treatment modifications, are more common on D-penicillamine than on zinc. Contrary to the group on D-penicillamine, none of our patients on zinc needed to change therapy because of side effects.

At variance with our data and those of others [8-16], the efficacy of zinc treatment for WD was recently questioned [17,18]. Several factors may be evoked to explain this discordance. Most of the patients evaluated by Weiss et al. started therapy in the adult age, when obviously the liver expression of WD is more severe. In the subset of patients classified as non-responders, they did not include patients who died or who underwent liver transplantation (10 under chelator versus 4 on zinc and 1 on combination therapy). Another important difference is that zinc was given as second-line therapy in their patients with liver disease. Thus, 11/14 patients that had hepatic failure on zinc after a median of 2.7 years (range: 0.67-18.34) of treatment had already received D-penicillamine for a median of 12.31 years (range: 0.27-33.59), and the outcome of these patients on chelator therapy was not reported. Therefore, the clinical status of these patients at the time of starting zinc therapy was not well defined. Moreover, although Weiss et al. reported that liver enzymes normalized in zinc non-responders after reintroduction of a chelating agent (rescue therapy), they did not provide information about rescue therapy. In contrast to these results, liver disease did not worsen in any of our long-term responders to D-penicillamine after switching to zinc monotherapy.

Again, we applied very stringent criteria to identify responders to therapy. In the Weiss’ study, the ranges of ALT and GGT in the responders at the end of the study were very wide (10–226 UI/L and 10–178 UI/L, respectively) and included values more than twice the upper limit of normal. In addition, while we evaluated zinc monotherapy for a median follow-up of 9.3 years, Weiss et al. did not report details about patients treated with zinc alone, apart from stating that the median duration of zinc therapy was 2.7 years in non-responders to zinc.

A major problem with WD treatment is that patients must comply strictly to therapy because low compliance or treatment discontinuation is associated with a high risk of hepatic decompensation that may even require liver transplantation [2]. Treatment compliance was not assessed by Weiss et al. They reported a lack of significant differences in serum and urinary zinc values between their responders and non-responders to zinc therapy; however, they did not report details about urinary copper levels. Consequently, they may have misjudged treatment adherence. It is noteworthy that the patients of Linn et al. that worsened under zinc therapy were later discovered not to have adhered to treatment based on their urinary copper levels [18]. In our study, the evaluation of urinary copper levels revealed an irregular intake of the drug in one of two non-responders to zinc who had appropriate serum and urinary zinc levels**.** Another important finding of our study is that urinary copper excretion does not represent a reliable efficacy parameter on penicillamine therapy, because some responders persistently maintain urinary copper level higher than 500 mcg/day. On the other hand, urinary copper excretion remains useful for evaluating the adherence to treatment in nonresponders to penicillamine and guiding the shift to zinc monotherapy in patients initially treated with penicillamine [2,4]. It is important to note that treatment failure was more frequent in our patients on combination therapy than in patients on other regimens, and that it was invariably linked to poor compliance. Given this result and the fact the efficacy of combination therapy is not yet well proven, the latter strategy should probably be avoided.

## Conclusions

In conclusion, our study addresses the challenge of how to treat WD patients with mild liver disease, diagnosed in childhood. In fact, we demonstrate that zinc monotherapy is effective in controlling WD-related liver disease both as first-line and as maintenance treatment in patients with mild liver disease. Its efficacy was evaluated in a large homogeneous cohort of WD patients diagnosed in childhood and followed for a long period. This study raises the possibility of treating, when indicated, WD patients with an effective and safe drug as zinc.

## Availability of supporting data

The data sets supporting the results of this article are included within the article and its additional files.

## Abbreviations

WD: Wilson’s disease; ATP7B: ATPase, Cu++ transporting beta polypeptide; KF: Kayser-Fleisher; ALT: Alanine aminotransferase; AST: Aspartate aminotransferase; γ-GT: γ-glutamyltransferase.

## Competing interests

The authors declare that they have no financial or non-financial competing interests.

## Authors’ contributions

Dr. GR conceptualized and designed the study, carried out analyses and interpretation of data, drafted the initial manuscript, and approved the final manuscript as submitted. Dr. FDD conceptualized and designed the study, carried out analyses and interpretation of data, drafted the initial manuscript, and approved the final manuscript as submitted. Dr. MIS designed the study, collected, analyzed, and interpreted the data and was responsible for drafting and revising the manuscript. Prof. PV designed the study, collected and interpreted the data, revised the manuscript, and approved the final version of the manuscript. Prof. RI conceptualized and designed the study, coordinated and supervised data collection and interpretation, drafted the initial manuscript, and approved the final manuscript as submitted. All authors read and approved the final version of the article, including the authorship list.
